# A Case of Starch Overload in Young Dairy Heifers: A Physiological and Nutritional Point of View

**DOI:** 10.3390/vetsci13040319

**Published:** 2026-03-26

**Authors:** Tommaso Danese, Emanuela Valle, Martina Lamanna, Riccardo Colleluori, Giovanni Buonaiuto, Isa Fusaro, Damiano Cavallini

**Affiliations:** 1 Department of Veterinary Sciences, University of Turin, Largo P. Braccin 2, 10095 Grugliasco, Torino, Italy; tommaso.danese@unito.it (T.D.); emanuela.valle@unito.it (E.V.); 2 Department of Veterinary Medical Sciences, University of Bologna, Via Tolara di Sopra 50, 40064 Ozzano dell’Emilia, Bologna, Italy; riccardo.colleluori2@unibo.it (R.C.); giovanni.bonaiuto@unibo.it (G.B.); damiano.cavallini@unibo.it (D.C.); 3 Department of Veterinary Sciences, University of Teramo, SP18, 64100 Teramo, Italy; ifusaro@unite.it

**Keywords:** dairy heifers, starch overload, diarrhea, rumen development, growth rate, feeding management

## Abstract

In order to guarantee sufficient growth, digestive stability, and long-term productivity in dairy heifers, proper nutritional management is crucial both before and after weaning. This case study assesses the impact of dietary modifications on growth performance and digestive parameters in commercial settings and details a field observation of concentrate overload in young Holstein heifers. From 77 to 165 days of age, the body weight (BW), average daily gain (ADG), body condition score (BCS), feed intake, and fecal characteristics of 15 calves were monitored. Infectious and parasitic causes of diarrhea were ruled out by fecal examinations. Ad libitum concentrate feeding resulted in low fecal scores with undigested grain particles and acidic smell, starch intake exceeding requirements, and concentrate intake reaching up to 6 kg as fed head×day. The BCS gradually rose, and ADG peaked at 1.64 kg/day. Forage intake increased, fecal consistency improved, and ADG stabilized after restricting concentrate allowance to 2.5% of BW. These results underline the significance of controlling starch intake and concentrate allowance to avoid excessive growth and digestive disorders in developing dairy heifers, and they support a nutritional basis for the observed digestive imbalance.

## Simple Summary

To become healthy and productive cows, dairy heifers need to grow at the right pace. However, excessive intake of concentrate feeds rich in starch can lead to digestive problems and excessive weight gain. This case report describes a group of young dairy heifers on a commercial farm showing loose manure and unusually rapid growth associated with high concentrate intake. On-farm monitoring and laboratory analyses suggested that excessive starch intake contributed to digestive imbalance. When concentrate allowance was restricted and forage intake increased, fecal consistency improved and growth rate stabilized. These observations highlight the importance of balancing concentrate and forage in heifer diets and of monitoring growth and fecal characteristics to detect nutritional imbalances early. Proper feeding management during this stage can help support digestive health, prevent excessive fat deposition, and promote better long-term productivity and animal welfare.

## 1. Introduction

The management of dairy calves during the early stages of life represents a critical component of replacement heifer production systems, both from an economic and biological perspective. Feeding strategies implemented during the pre-weaning and immediate post-weaning phases have long-term implications for growth performance, metabolic health, mammary development, and future milk yield [1,2]. Because of this, the importance of calf nutrition in determining lifetime productivity and farm profitability is becoming more widely acknowledged.

During the first few weeks of life, newborn calves, which are functionally monogastric animals, mainly rely on milk digestion. The development of rumen structure, microbial colonization, and metabolic adaptation to solid feed intake are all part of the slow and intricate process of becoming a functional ruminant [3]. The introduction of starter feed, which promotes microbial fermentation and the production of volatile fatty acids (VFAs), especially butyrate and propionate, which support rumen epithelial development, is what causes this shift [4,5,6]. At the same time, adequate physical fiber intake plays a fundamental role in supporting rumen motility and muscular development, while helping to regulate fermentation patterns. The balance between fermentable carbohydrates and structural fiber is therefore essential to ensure proper rumen maturation and digestive stability. If this balance is disrupted, especially through excessive intake of rapidly fermentable carbohydrates such as starch, the developing digestive system may not be able to adequately regulate fermentation processes [7].

Under practical farm conditions, management of replacement calves may receive less attention compared with lactating animals, as they do not generate immediate economic returns. However, suboptimal feeding strategies during this phase can have significant long-term consequences, including altered growth trajectories, excessive fat deposition, impaired mammary gland development, and reduced milk production in later life [8,9].

To prevent unintentional metabolic and developmental imbalances, it is crucial to closely monitor growth parameters and nutritional intake.

Although there are well-established guidelines for calf feeding and rumen development, there is still little evidence of field cases where young heifers raised in commercial settings experience noticeable physiological and digestive problems as a result of poor nutritional management. Specifically, detailed descriptions of the effects of uncontrolled concentrate intake during the transition from pre-ruminant to ruminant stages are rare. The aim of this case report is to describe a field-observed condition of concentrate overload in young dairy heifers and to evaluate its effects on growth performance, fecal characteristics, and indicators of digestive imbalance under commercial farm management.

## 2. Materials and Methods

### 2.1. Animals

The case was conducted in a commercial Holstein dairy herd located in northeast Italy, within the Parmigiano Reggiano cheese production area. The herd consisted of approximately 150 lactating cows. The farm was visited during a nutritional consultancy advised by the farm’s referring veterinarian.

A group of 15 pre-weaning female Holstein calves (future dairy heifers) was selected for monitoring based on the presence of abnormal growth patterns (above expected average daily gain) and altered fecal consistency (persistently low fecal scores and presence of undigested particles). Animals were selected during a veterinary nutritional consultation, and inclusion criteria were based on clinical observation of rapid growth, increased body condition score, and signs of digestive imbalance in the absence of evident infectious disease. Calves were housed in multiple concrete pens bedded with abundant straw. Weaning was routinely performed at approximately 3 months of age, according to farm management practices.

Before and after weaning, calves were fed ad libitum concentrate and hay. The high concentrate intake observed in this group was not part of a planned feeding strategy, but reflected routine farm management practices, where concentrate was offered ad libitum without adjustment for body weight or developmental stage. Milk allowance before weaning was maintained at 3 L head×day until nutritional interventions were applied. All animals were managed according to standard commercial practices, and no experimental procedures were imposed.

### 2.2. Feeds and Feed Analysis

During the initial evaluation, calves were fed a commercial concentrate ad libitum (Feed A) and first-cut hay produced on-farm. Feed A was composed of corn, hulled soybean meal, wheat bran, toasted soybeans, dried beet pulp, sugarcane molasses, soybean oil, calcium carbonate, dicalcium phosphate, and sodium chloride. The declared chemical composition included 20% crude protein (CP), 5% crude fiber (CF), 5% ether extract (EE), and 7% ash. Vitamin, mineral, and additive contents were provided by the manufacturer and included live yeast (*Saccharomyces cerevisiae*, 1 × 10^9^ CFU).

During the monitoring period, two feeding management interventions were implemented. At approximately 113–119 days of age, concentrate (Feed A) was temporarily restricted to three meals of approximately 1 kg each. Later, from approximately 134 days of age onward, concentrate allowance was restricted to 2.5% of estimated body weight. Feed B consisted of corn, hulled sunflower meal, wheat bran, corn gluten meal, sunflower seed by-products, wheat middlings, hulled soybean meal, calcium carbonate, sugarcane molasses, and sodium chloride. The declared composition of Feed B included 19% CP, 10% CF, 4% EE, and 6.6% ash.

The chemical compositions of Feed A and B were determined through laboratory analysis, and the results are reported in Table 1. Nutrient composition of the first-cut hay is reported in Table 2.

All feed samples were analyzed for dry matter (DM), CP, EE, neutral detergent fiber (NDF), acid detergent fiber (ADF), acid detergent lignin (ADL), ash, and starch using a TANGO FT-NIR spectrometer (Bruker Optics GmbH, Ettlingen, Germany), according to previous reports [10,11].

### 2.3. Animal Measurement and Monitoring

Calves were monitored weekly by the same trained operator throughout the pre- and post-weaning period, from the day of inclusion (77 days old) to 165 days of life (11 monitoring time points), to ensure consistency in data collection. Body weight, wither height, and chest girth were recorded to evaluate growth performance and body development. Body weight (BW) was measured using a calibrated electronic scale, while wither height and chest girth were measured using standard measuring tools and anatomical reference points to ensure repeatability. Average daily gain (ADG) was calculated for each monitoring interval.

Body condition score (BCS) was assessed by the same evaluator to minimize inter-observer variability, using a 5-point scale [12], to evaluate fat deposition and overall body condition.

Feed intake was estimated daily by recording the amount of feed offered and weighing refusals, and values were expressed as average intake per animal. Milk intake was recorded daily until weaning. Digestive function was evaluated by fecal consistency scoring [12] and by wet sieving of fecal samples, as described by [13], to assess the presence of undigested feed particles.

To exclude infectious and parasitic causes of diarrhea, individual fecal samples were collected directly from the rectum by the attending veterinarian using disposable gloves, ensuring minimal contamination. Samples were placed in sterile containers and analysed shortly after collection.

Coprological examinations were performed by a veterinary diagnostic laboratory using standard flotation techniques for the detection of coccidia and Cryptosporidium spp. In addition, commercial antigen detection tests were used to identify rotavirus, coronavirus, *Cryptosporidium* spp., and *Escherichia coli* F5 (K99), following the manufacturer’s instructions.

All monitoring procedures were conducted under routine farm conditions without experimental manipulation, ensuring that observations reflected real-world management practices.

## 3. Results

### 3.1. Baseline Conditions and Initial Clinical Findings

At the beginning of the monitoring period (77 ± 2 days of life), the 15 pre-weaning calves had an average body weight of 101 kg, with a wither height of 92 cm and a chest girth of 110 cm (Table 3). Milk intake was 3 L head×day. The average fecal score was 1.5, indicating loose fecal consistency (Table 4).

### 3.2. Growth Performance and Body Condition Development

Throughout the monitoring period, calves exhibited a rapid growth rate. Average daily gain ranged from 0.92 to 1.64 kg/day depending on the interval between consecutive monitoring measurements, with progressive increases in body weight, average daily gain and BCS (Table 4).

BCS increased over time, reaching values close to or exceeding 2.8 during the later stages of the monitoring period, particularly during phases characterized by high concentrate intake.

### 3.3. Feed Intake Patterns and Dietary Changes

Concentrate intake progressively increased, reaching approximately 3.4–3.8 kg as fed head×day, whereas forage intake remained limited (Table 4).

At approximately 113–119 days of age, a temporary restriction of Feed A to three meals of approximately 1 kg each was applied. This intervention resulted in increased forage intake and an improvement in fecal score, whereas milk reduction and subsequent weaning did not produce appreciable changes in fecal consistency.

At approximately 123–133 days of age, Feed A was replaced by Feed B, which was initially offered ad libitum. During this phase, concentrate intake further increased, reaching values up to 6 kg as fed head×day, while forage intake markedly decreased.

From approximately 134 days of age onward, concentrate allowance was restricted to 2.5% of estimated BW. Following this intervention, concentrate intake decreased to approximately 4.5–4.9 kg as fed head×day, and forage intake increased.

### 3.4. Fecal Characteristics and Digestive Findings

During the initial and intermediate phases of the monitoring period (77–113 days of age), characterized by ad libitum concentrate feeding, loose fecal consistency persisted, with fecal scores ≤ 1.5. Wet sieving performed during this phase revealed the presence of undigested corn particles and mucus in the feces.

During the temporary restriction of Feed A (approximately 113–119 days of age), fecal consistency showed a partial improvement.

During the subsequent phase in which Feed B was offered ad libitum (approximately 123–133 days of age), fecal consistency deteriorated again, and an acidic odor of the feces was noted.

From approximately 134 days of age onward, following restriction of concentrate allowance to 2.5% of estimated BW, fecal consistency progressively improved, with fecal scores increasing to values ≥ 2.5 and a greater proportion of fibrous material visually detectable in the feces.

A temporal association between fecal score and estimated dietary starch concentration across the monitoring period is reported in Table 5.

### 3.5. Health Screening

Coprological examination and antigenic testing were negative for coccidia, *Cryptosporidium* spp., rotavirus, coronavirus, and Escherichia coli F5 (K99).

## 4. Discussion

The present case report describes a condition of concentrate overload in young dairy heifers, and its relation to increased growth rate, persistently low fecal score, and impaired digestive efficiency in the absence of infectious or parasitic diseases. The temporal association between high concentrate intake, elevated dietary starch content, and altered fecal characteristics strongly supports a nutritional origin of the observed disorder [7]. High levels of concentrate supplementation can alter feeding behavior and reduce forage intake due to substitution effects and changes in ruminal fermentation dynamics [14]. However, nutritional strategies based on concentrate restriction must also be carefully balanced to ensure adequate energy supply and maintain appropriate growth rates: maintaining an adequate forage-to-concentrate ratio represents key management strategies to support digestive stability and optimal development of replacement heifers [15].

In this case, weaning was performed at approximately three months of age, in accordance with the farm’s routine management. Although age-based weaning strategies are commonly adopted, optimal weaning management should consider additional parameters such as body weight and concentrate intake [16], adjusted based on breed and environmental factors. For large-breed calves, a minimum intake of approximately 0.9 kg of concentrate DM for at least three consecutive days has been recommended as a criterion for weaning [3]. In the present case, weaning decisions were driven primarily by age, and this may have contributed to an early exposure of the calves to high levels of fermentable carbohydrates.

When the present data were compared with available reference values, BW and concentrate intake, between approximately 11 and 20 weeks of age, were within the ranges reported for Holstein heifers. However, differences emerged when growth dynamics were evaluated over time. According to some conservative growth targets proposed [17], a BW of approximately 175 kg at six months of age is recommended to limit excessive prepubertal fat deposition. In the present case, this threshold was reached and exceeded before six months of age, with heifers weighing approximately 200 kg at around 5.5 months of age.

From a nutritional standpoint, NRC recommendations [18] provide a broader target BW of approximately 200 kg for Holstein heifers between 3 and 6 months of age, which was also reached earlier than expected in this case. In addition, ADG during the late prepubertal phase markedly exceeded commonly recommended values (1.64 vs. approximately 1.0 kg/day), and dry matter intake at around six months of age (7.4 kg/day) was higher than reference values [17,18].

Similarly, BCS progressively increased, approaching values associated with early fat deposition. Excessive growth rates and over conditioning during the prepubertal phase have been associated with impaired mammary gland development and reduced future milk production, likely mediated by alterations in endocrine signaling and mammogenic hormone secretion [19]. Therefore, the progressive increase in BCS observed in this case raised long-term concerns beyond digestive health alone. These data suggest that the issue was not the absolute BW achieved, but rather the timing and velocity of growth, with target BW being reached earlier than recommended during the prepubertal phase. From a physiological perspective, accelerated growth combined with increased BCS is associated with enhanced adipose tissue deposition within the mammary gland, which may compromise the development of functional parenchymal tissue. This process can negatively affect future milk production potential, as excessive fat accumulation during the prepubertal period has been linked to reduced mammary secretory capacity. A consistent relationship between dietary starch intake and fecal score was observed throughout the monitoring period. Low fecal scores and the presence of mucus and undigested grain particles were recorded during phases characterized by ad libitum concentrate feeding and estimated dietary starch levels exceeding 20% DM. In contrast, fecal consistency progressively normalized following concentrate restriction, when dietary starch content fell within recommended ranges. These findings are consistent with nutritional guidelines indicating optimal starch concentrations between 15 and 20% DM for growing calves and heifers, as reported in Table 6 [18].

From a physiological perspective, excessive intake of rapidly fermentable carbohydrates during the transition from pre-ruminant to functional ruminant status may overwhelm the adaptive capacity of both the rumen and the hindgut. In calves that are still developing ruminal absorptive and buffering mechanisms, high starch intake can increase the rate of fermentation and volatile fatty acid production beyond epithelial absorption capacity, leading to a decline in ruminal pH [20,21]. The source and fermentability of dietary starch can markedly influence rumen fermentation patterns, rumination activity, and overall digestive stability in growing ruminants. Differences in starch degradability among feed ingredients may also affect chewing activity and rumination behavior. Highly fermentable starch sources may increase the risk of fermentation imbalances when not adequately balanced with dietary fiber, highlighting the importance of controlling starch supply and its fermentability in heifer feeding strategies [22].

In pre-ruminant or early-transition calves, the intestinal mucosa is not fully adapted to large post-ruminal starch loads. Excess substrate reaching the hindgut undergoes rapid microbial fermentation, resulting in lactic acid accumulation, osmotic water influx, and increased luminal acidity, contributing to nutritional diarrhea through osmotic imbalance and mucosal irritation [23].

Moreover, both ruminal and hindgut acidosis have been associated with epithelial damage, inflammation, and translocation of lipopolysaccharides, contributing to systemic health challenges [24]. Although the precise site of acidosis could not be determined under commercial farm conditions [25], the acidic odor of feces and the marked improvement following concentrate restriction are consistent with carbohydrate-induced digestive acidosis [26].

In this context, fecal evaluation proved to be a practical and informative on-farm diagnostic tool: changes in fecal consistency, odor, and particle size composition closely mirrored dietary modifications and provided immediate feedback on digestive balance. Low fecal scores were observed exclusively during periods of high starch intake, whereas optimal fecal consistency was achieved only after concentrate allowance was limited and forage intake increased. These observations highlight the value of routine fecal assessment, where fecal particle size confirms its utility as a mirror of a healthy gastrointestinal tract and indicator of ration adequacy in growing heifers; in this context, routine fecal evaluation may represent an early warning tool, allowing practitioners to identify excessive fermentable carbohydrate intake before overt metabolic or developmental consequences become evident.

This study has several limitations, including the absence of a control group and the limited number of animals enrolled, restricting the generalizability of the findings. However, the present work was designed as a field case report conducted during a veterinary nutritional consultation under commercial farm conditions, and therefore, the inclusion of a control group was not feasible.

Overall, this case emphasizes the importance of regulating concentrate intake in young dairy heifers to prevent digestive disorders, excessive growth rates, and over conditioning during critical developmental stages. Under commercial conditions, concentrate restriction based on BW proved to be an effective and practical approach to restoring digestive balance and controlling growth rate in replacement heifers [15]. As technology continues to advance, precision livestock farming (PLF) systems are increasingly being implemented to support dairy farmers, technicians, and veterinarians in the accurate quantification and evaluation of management parameters [27,28]. From a broader herd management perspective, the condition described in this case reflects a mismatch between nutritional supply and the physiological capacity of the developing animal, which may not be immediately evident under routine farm conditions. Early detection of such imbalances relies heavily on the systematic monitoring of key indicators, including growth rate, body condition score, feed intake patterns, and fecal characteristics. In this context, the integration of objective and continuous monitoring approaches could enhance the ability to identify subtle deviations from optimal developmental trajectories. Precision Livestock Farming (PLF) technologies, which enable the collection of real-time behavioral and physiological data, may represent a valuable complementary tool to traditional on-farm assessments, particularly in supporting early warning systems for nutritional and metabolic disorders. However, their application in young heifers remains limited, and further research is needed to validate their effectiveness and practical implementation under commercial conditions [29,30].

## 5. Conclusions

This case report highlights how excessive concentrate intake and elevated dietary starch levels can impair digestive function and promote excessive growth in young dairy heifers under commercial conditions. Nutritional diarrhea associated with concentrate overload can be effectively prevented through a limited number of complementary strategies, including reducing total starch intake and the use of less rapidly fermentable carbohydrate sources, together with the promotion of adequate roughage intake through ration formulation or concentrate restriction.

When formulating rations for growing heifers, the primary objective should be to achieve appropriate BW targets while avoiding overconditioning during critical developmental stages. Regular monitoring of body weight, wither height, chest girth, and BCS is essential to assess growth dynamics, while routine evaluation of fecal consistency provides a practical and effective on-farm tool to detect dietary imbalances. Together, these approaches allow timely nutritional adjustments that support both digestive health and optimal long-term performance of replacement heifers.

## Figures and Tables

**Table 1:** Chemical composition of Feed A and Feed B.

Item	Feed A	Feed B
DM ^1^, % as fed	87.01	88.88
CP, %DM	23.8	21.38
EE, %DM	4.85	6.48
NDF, %DM	16.27	28.57
ADF, %DM	7.55	16.91
ADL, %DM	1.33	4.24
Ash, %DM	7.30	8.81
Starch, %DM	33.92	25.10

^1^ DM = dry matter, CP = crude protein, EE = ether extract, NDF = neutral detergent fiber, ADF = acid detergent fiber, ADL = acid detergent lignin.

**Table 2:** Chemical composition of the first cut hay.

Item ^1^	First Cut Hay
DM, % as fed	90.88
CP, % DM	8.56
EE, % DM	1.35
aNDFom, % DM	64.18
ADF, % DM	42.96
ADL, % DM	6.69
Ash, % DM	5.24
Sugars, % DM	8.36

^1^ DM = dry matter, CP = crude protein, EE = ether extract, aNDFom = amylase-treated ash-corrected neutral detergent fiber with addition of sodium sulphite, ADF = acid detergent fiber, ADL = acid detergent lignin.

**Table 3:** Baseline age (days; d), wither height (cm), and chest girth (cm) of the monitored pre-weaning calves across the monitoring period.

Days of Life (d)	Wither Height (cm)	Chest Girth (cm)
77	92	110
84	93	112
89	95	114
91	96	117
98	98	120
113	104	132
114	104	132
119	105	134
123	106	138
126	106	138
133	107	140
134	108	143
140	108	143
141	109	145
147	110	148
148	111	148
154	112	148
155	112	150
161	113	153
162	113	153
165	113	153

**Table 4:** Baseline age (days; d), body weight (kg), average daily gain (ADG; kg), body condition score (BCS), fecal score, milk, forage and concentrate intakes (l and kg DM, respectively) of the monitored pre-weaning calves across the monitoring period.

Days of Life (d)	Body Weight (kg)	ADG (kg)	BCS	Fecal Score	Milk Intake (L)	Forage Intake (kg DM)	Concentrate Intake (kg DM)
77	101	1.07	2.50	1.5	3.0	0.36	1.41
84	109	1.07	2.50	1.5	3.0	0.36	1.41
89	114	1.07	2.36	1.5	2.0	0.73	2.82
91	116	1.07	2.36	1.5	0.0	0.73	2.82
98	124	1.05	2.36	1.0	0.0	0.82	3.17
113	139	1.05	2.46	1.0	0.0	0.73	3.34
114	140	0.92	2.46	2.0	0.0	2.27	2.64
119	145	0.92	2.46	2.0	0.0	2.27	2.64
123	149	0.92	2.50	2.0	0.0	0.36	4.66
126	151	0.92	2.50	2.0	0.0	0.36	4.66
133	157	1.14	2.50	1.5	0.0	0.36	5.28
134	159	1.14	2.75	2.5	0.0	2.27	3.96
140	167	1.14	2.75	2.5	0.0	2.27	3.96
141	168	1.57	2.75	3.0	0.0	2.27	4.14
147	175	1.57	2.75	3.0	0.0	2.27	4.14
148	176	1.57	2.86	3.0	0.0	2.36	4.31
154	189	1.57	2.86	3.0	0.0	2.36	4.31
155	191	1.64	2.86	3.0	0.0	2.45	4.66
161	200	1.64	2.86	3.0	0.0	2.45	4.66
162	202	1.64	2.86	3.0	0.0	2.45	4.93
165	207	1.64	2.86	3.0	0.0	2.45	4.93

**Table 5:** Temporal association (days; d) between fecal score and estimated dietary starch concentration (% DM) across the monitoring period.

Days of Life (d)	Fecal Score	Starch Intake (%DM)
77	1.5	27
84	1.5	27
89	1.5	27
91	1.5	27
98	1	27
113	1	28
114	2	18
119	2	18
123	2	23
126	2	23
133	1.5	23
134	2.5	16
140	2.5	16
141	3	16
147	3	16
148	3	16
154	3	16
155	3	16
161	3	16
162	3	17
165	3	17

**Table 6:** Nutrient concentrations (DM basis) requirements for Holstein cattle at varying stages of lactation and ages of maturity (adapted from [18]).

	Growing Calves and Heifers (Age, Days)					
Parameter	30 d	100 d	225 d	350 d	475 d	600 d
BW (kg)	65	120	230	330	420	530
Growth rate (kg/d)	0.7	0.7	0.9	0.8	0.7	0.9
Dry matter intake (kg/d)	1.4	3.9	6.6	8.5	9.8	11.0
ME (MJ/kg)	3.68	2.26	2.09	1.95	1.92	2.12
NE (MJ/kg)	-	-	-	-	-	-
Rumen-degradable protein (%)	-	10.0	10.0	10.0	10.0	10.0
Rumen-undegradable protein (%)	-	6.6	4.4	2.6	1.7	2.7
Crude protein (%)	21.0	16.6	14.4	12.6	11.7	12.7
Metabolizable protein (%)	16.5	9.5	8.1	6.8	6.1	14.0
NDF min (%)	-	25–33	25–33	25–33	25–33	25–33
Forage NDF min (%)	-	19–25	19–25	19–25	19–25	19–25
Starch max (%)	-	15–20	15–20	15–20	15–20	15–20
Ca (%)	0.59	0.78	0.58	0.44	0.37	0.39
P (%)	0.45	0.32	0.26	0.21	0.18	0.19
Mg (%)	0.15	0.14	0.12	0.12	0.12	0.10
K (%)	1.00	0.51	0.52	0.54	0.56	0.60
Na (%)	0.35	0.17	0.16	0.16	0.15	0.16
Cl (%)	0.28	0.14	0.14	0.13	0.13	0.13
S (%)	-	0.20	0.20	0.20	0.20	0.20
DCAD (mEq/kg min)	-	39	42	45	50	60
Cu (mg/kg)	5	16	16	15	15	17
Co (mg/kg)	-	0.20	0.20	0.20	0.20	0.20
I (mg/kg)	0.78	0.69	0.58	0.54	0.53	0.54
Fe (mg/kg)	90	61	46	32	24	28
Mn (mg/kg)	50	49	44	40	38	43
Se (mg/kg)	0.3	0.3	0.3	0.3	0.3	0.3
Zn (mg/kg)	70	47	41	36	34	35
Vitamin A (IU/kg)	5218	3390	3829	4265	4698	5288
Vitamin D (IU/kg)	1518	924	1044	1163	1281	1442
Vitamin E (IU/kg)	86	49	56	62	68	77

## Data Availability

The original contributions presented in this study are included in the article. Further inquiries can be directed to the corresponding author(s).
